# HIV transmission should be decriminalized: HIV prevention programs depend on it

**DOI:** 10.1186/1742-4690-5-108

**Published:** 2008-12-01

**Authors:** Mark A Wainberg

**Affiliations:** 1 McGill University AIDS Centre, Jewish General Hospital, Montreal, Quebec, Canada

## Abstract

Whenever there is a sensational criminal case involving HIV transmission, the media cover it with far more gusto than they usually devote to scientific advances in the field. For example, a murder trial is now taking place in Canada involving a man who has been accused of sexually transmitting HIV to 11 different women, two of whom have died of their infections. Moreover, it is alleged that the accused perpetrator deliberately withheld from these women the fact that he was HIV-positive and that he refused to use a condom during intercourse. Notwithstanding that the suspect is possibly psychopathic and uncaring, or possibly of low intelligence and unable to assess the consequence of his actions, most people probably hope that he is convicted, sentenced, and imprisoned for his acts. Furthermore, most people probably wish for the criminal justice system to pursue these cases with vigour. In fact, however, people should understand that such legal action, and the willingness of the courts to hear these cases, will only weaken the global battle against HIV transmission.

## Editorial

In the 25 years since the discovery of HIV [1,2], there have been a number of such cases. The fact is that they have probably done more harm than good, even when it has been possible to prove wilful transmission. In almost all such instances, defence lawyers have seized upon the discredited notion that HIV may not cause AIDS in the first place, to make their arguments as was recently seen in the Parenzee case in Australia [3]. Because these cases have often attracted widespread coverage in the press, the result has usually been to cause confusion as to the harmful nature of HIV and to give the so-called HIV denialists a platform from which to promulgate their views [3].

More important though is that the consideration of being potentially charged with wilful HIV transmission may be a significant deterrent to being tested for HIV infection in the first place. After all, an individual who does not know that he is HIV positive cannot logically be accused of its transmission. This leads to two major negative consequences.

The first is that failure to identify as many HIV positive people as possible will lead to higher rates of HIV spread than would otherwise occur. Multiple studies have now shown that individuals who are informed that they are HIV positive will commonly desist from high-risk sexual behaviour in an effort to protect sexual partners from the virus, but may not do so if they are unaware of their own status. This point cannot be over-emphasized, since research has also revealed that as many as 50% of all new HIV transmissions are attributable to people who may themselves only be recently infected [4,5]. One reason for this is that levels of virus in the blood and sexual fluids are usually very high for about a 6-month period following infection.

The second negative consequence of delayed testing is that many HIV-infected persons may not become diagnosed until at least several years after infection, thus giving the virus an opportunity to replicate throughout this time and cause significant, often irreversible, damage to the immune system. This may sometimes result in life-threatening infections that could probably have been prevented had these people been diagnosed earlier and commenced therapy sooner with anti-HIV drugs. There are also concerns that failure to initiate anti-HIV therapy early may leave people more vulnerable to a variety of cancers than the population at large.

The other major benefit of an earlier initiation of anti-HIV therapy is that it lowers the amounts of virus in both blood and sexual fluids, thereby rendering people far less infectious for their sexual contacts [6]. Indeed, some groups have now proclaimed that persons whose viral replication is fully suppressed by antiviral treatment need no longer use condoms or take other precautions when having sexual relations with regular partners [7]. Although health authorities have not endorsed this controversial recommendation, its very existence underlines that appropriate use of anti-HIV drugs will not only improve the health of infected persons but may also have benefits for HIV spread and public health.

All of the above constitute grounds for advocating frequent testing for individuals who might be at risk of contracting HIV, in the hope of attaining earlier diagnoses. Yet, the risk of being accused of a crime in regard to HIV transmission, alongside the stigma of being identified as HIV positive, constitute significant deterrents for many people in agreeing to be tested to begin with.

How can society resolve this problem, while not, in effect, encouraging sexual promiscuity and risk behaviour? First, we need to recognize that the current criminalization of HIV transmission is not doing any good and, probably acts as a deterrent to HIV testing, thereby, in effect, promoting HIV transmission by people who do not know or don't want to know that they are infected. We also need to accept that having sexual relations involves a personal responsibility to know one's partner on much more than a superficial level.

Finally, let's not confuse the issue of HIV testing and personal responsibility for consensual sexual relations with that of HIV transmission by rapists or other perpetrators of crime. Clearly, people who sexually assault others and force them into non-consensual sex should continue to be charged and tried under the law. Probably, as well, a person who throws contaminated blood or needles at someone should be charged with assault, since their intent was most likely to cause harm, notwithstanding that any resultant skin contact with such blood would be extremely unlikely to result in HIV transmission. But, the putative crime in such cases would be assault rather than intent to transmit HIV.

If the evidence against the accused in the Canadian case is upheld in court, this will substantiate that he is indeed the unsavoury, irresponsible individual that the prosecutors have made him out to be. But, let's also recognize that our policies regarding criminalization of HIV transmission are having the opposite effect of those that were intended and fix things in order to do a much better job in regard to overall public health. On World AIDS day 2008, this is a topic worthy of further thoughtful consideration.
